# The amino acid metabolism is essential for evading physical plasma-induced tumour cell death

**DOI:** 10.1038/s41416-021-01335-8

**Published:** 2021-03-25

**Authors:** Rajesh Kumar Gandhirajan, Dorothee Meyer, Sanjeev Kumar Sagwal, Klaus-Dieter Weltmann, Thomas von Woedtke, Sander Bekeschus

**Affiliations:** 1 ZIK plasmatis, Leibniz Institute for Plasma Science and Technology (INP), Greifswald, Germany; 2 Institute for Hygiene and Environmental Medicine, Greifswald University Medicine, Sauerbruchstr, Greifswald, Germany

**Keywords:** Physics, Cancer metabolism

## Abstract

**Background:**

Recent studies have emphasised the important role of amino acids in cancer metabolism. Cold physical plasma is an evolving technology employed to target tumour cells by introducing reactive oxygen species (ROS). However, limited understanding is available on the role of metabolic reprogramming in tumour cells fostering or reducing plasma-induced cancer cell death.

**Methods:**

The utilisation and impact of major metabolic substrates of fatty acid, amino acid and TCA pathways were investigated in several tumour cell lines following plasma exposure by qPCR, immunoblotting and cell death analysis.

**Results:**

Metabolic substrates were utilised in Panc-1 and HeLa but not in OVCAR3 and SK-MEL-28 cells following plasma treatment. Among the key genes governing these pathways, ASCT2 and SLC3A2 were consistently upregulated in Panc-1, Miapaca2GR, HeLa and MeWo cells. siRNA-mediated knockdown of ASCT2, glutamine depletion and pharmacological inhibition with V9302 sensitised HeLa cells to the plasma-induced cell death. Exogenous supplementation of glutamine, valine or tyrosine led to improved metabolism and viability of tumour cells following plasma treatment.

**Conclusion:**

These data suggest the amino acid influx driving metabolic reprogramming in tumour cells exposed to physical plasma, governing the extent of cell death. This pathway could be targeted in combination with existing anti-tumour agents.

## Background

Cancer is a devastating disease and the second cause of death in western societies. A particular trait of many types of cancers is their ability to evolve and become refractory to different treatment modalities, such as chemotherapy, radiotherapy and immunotherapy. To this end, novel treatment avenues are utterly needed to target tumours from multiple pathways simultaneously. Among many approaches being investigated in preclinical research, cold physical plasma has gained traction in translational research due to its inherent capacity to deposit many different ROS (reactive oxygen species) directly on the tumour tissue.^1^

Physical plasma is a partially ionised gas and multicomponent system. Plasmas expel ions, electrons, UV radiation, electric fields, and ROS to a different degree, depending on the type of plasma system and its settings being used.^2^ Plasma-derived ROS have been shown to limit tumour growth in several animal models, including, e.g. skin cancer,^3–5^ pancreatic cancer^6–8^ and colon cancer.^9,10^ As a mechanism of action, it is assumed that the ROS cause a redox imbalance in the tumour cells, leading to cell death while affecting some types of non-transformed cells to a lesser extent.^11^ Metabolic alterations and elevated levels of reactive oxygen species (ROS) are two characteristic hallmarks of cancer.^12^ The reciprocal relationship between metabolic and redox signalling is required for sustained growth and proliferation of cancer cells.^13^ Because of increased metabolic activity, tumour cells accumulate ROS, leading to increased oxidative stress.^14,15^ To cope with the oxidative stress, tumour cells adapt several ROS scavenging systems to maintain ROS levels below the toxic threshold to prevent oxidative damage.^16^

**Fig. 1:**
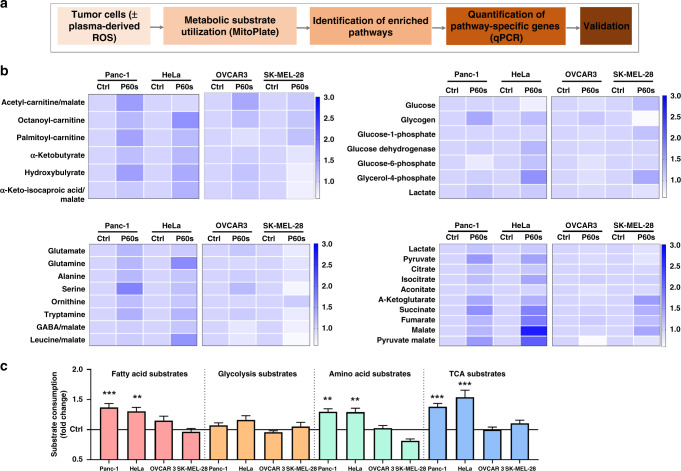
Substrate dependency of tumour cell lines following gas plasma treatment in vitro. **a** flow chart depicting the proposed study design; **b** heatmap of the metabolic substrate consumption of fatty acids, glycolysis, amino acid and TCA cycle in Panc-1, HeLa, OVCAR3, and SK-MEL-28 cells 3 h following plasma exposure; **c** fold changes in pathway-specific substrate consumption reveal Panc-1 and HeLa cells consumed predominantly fatty acids, amino acid and TCA substrates following plasma treatment (bars) normalised to each untreated cell line control (solid line). Data are mean ± SEM derived from two to three independent experiments with *p < 0.05, **p < 0.01 and *** *p* < 0.001. Statistical analysis was determined using two-tailed *t*-test.

Furthermore, ROS also leads to post-translational modifications (PTMs) on redox-sensitive enzymes, thereby influencing their function.^17^ This eventually leads to metabolic reprogramming and resistance to several therapeutics, resulting in poor prognosis in patients. We recently determined the intrinsic tumour cell resistance to plasma-induced cell death and identified a decisive role of the cystine/glutamate antiporter SLC7A11 (xCT).^18^ xCT plays a major role in glutathione biosynthesis and is closely linked to metabolic pathways in tumour cells.^19^ This enhanced biosynthetic activity is an essential feature of metabolic reprogramming in cancer as it supports the production of macromolecules (DNA, protein and lipid) mostly derived from amino acids.^20^ This led to the hypothesis that metabolic substrates/pathways could be harnessed by tumour cells that could counter plasma-based therapeutic interventions. In this study and using a two-step screening approach (Fig. 1a), we investigated the pathways and genes induced by plasma treatment in several tumour cell lines that might contribute towards metabolic reprogramming and extended survival.

## Methods

### Cell culture

The human tumour cell lines Panc-1 (CRL-1469; ATCC, Manassas, Virginia, USA), HeLa (ATCC CRM-CCL-2), MeWo (ATCC HTB-65), MaMel86a (CVCL A221), Miapaca2GR (ATCC CRM-CRL-1420), OVCAR3 (ATCC HTB-161) and SK-MEL-28 (ATCC HTB-72), as well as a non-tumorigenic human mesenchymal stem cell line (HMSC; PromoCell, Heidelberg, Germany), were grown in high glucose *Dulbecco*’*s* minimum essential medium (DMEM; Invitrogen, Karlsruhe, Germany) or Roswell Park Memorial Institute (RPMI1640; Invitrogen) medium in a humidified atmosphere at 5% CO_2_ and 37 °C. The medium was supplemented with 10% foetal bovine serum (FBS), 1% penicillin/streptomycin and 1% glutamine (Invitrogen).

### Plasma treatment and 2D imaging

Cells (1 × 10^4^/well) were plated in 96-well plates. Sixteen hours later, the medium was exchanged with RPMI medium supplemented with 2% FBS. Cells were then exposed for 60 s to cold physical plasma (P60s) or the argon gas alone (control). Cold physical plasma treatment was performed using the atmospheric pressure plasma jet kINPen operated at 1Mhz and two standard litres per min of argon gas (99.999% purity; Air Liquide, Hamburg, Germany) as outlined previously.^21^ The technical properties of the plasma jet were described in detail before.^22^ Twenty-four hours after plasma treatment, the cells’ metabolic activity was analysed by the addition of resazurin (100 µM; Alfa Aesar, Kandel, Germany) that NADPH reduces to the fluorescent resorufin. Fluorescence was acquired using a multimode plate reader (F200; Tecan, Männedorf, Switzerland) at *λ*_ex_ 535 nm and *λ*_em_ 590 nm. Viability was determined by sytox green staining (1 µM; Thermo Fisher Scientific, Hennigsdorf, Germany), a dye entering only cells with compromised membranes and that fluoresces upon binding to DNA. Analogously to endogenous glutathione levels (GSH-tracer, 5 µM; Tocris, Bristol, UK) and mitochondria membrane potential (TMRE, 1 µM; AAT Bioquest, Sunnyvale, California, USA), it was analysed using a live-cell high content imaging system (Operetta CLS; PerkinElmer, Hamburg, Germany) as described before.^23^ A digital phase contrast (DPC) channel was used for algorithm-driven cell segmentation to allow for fluorescence quantification within the dedicated imaging software (Harmony 4.9; PerkinElmer). Cells were incubated with the ASCT2 antagonist V9302 (10 µM; Sigma–Aldrich, Darmstadt, Germany) 2 h before plasma treatment, and the extent of toxicity was determined by sytox green staining.

### Spheroid assay

5 × 10^3^ Panc-1 cells were seeded in ultra-low affinity plates (PerkinElmer, Hamburg, Germany) for 48 h. The media was replaced with RPMI medium (2% FCS), and spheroids were plasma-treated for 60 s. Six hours later, the medium was replaced with an amino acid-free medium (EBSS with 2% FBS), supplemented with amino acids glutamine, tyrosine or valine (200 µM) along with respective controls. Seventy-two hours later, the spheroids were counterstained with Hoechst (10 µM; Sigma–Aldrich, Darmstadt, Germany) and sytox green (5 µM; Thermo Fisher Scientific, Hennigsdorf, Germany) for 1 h at 37 °C. Spheroid images were acquired using 50 stacks per well with a live-cell high content imaging system (Operetta CLS; PerkinElmer, Hamburg, Germany) and quantified using dedicated imaging software (Harmony 4.9; PerkinElmer).

### MitoPlate assay

For the MitoPlate assay, 1 × 10^6^ cells were seeded in RPMI medium (2% FBS) in 24-well plates (Sarstedt) and treated for 60 s with plasma. After 1 h of incubation, the cells were detached and added to a MitoPlate S-1 (Biolog, Taucha, Germany) containing 31 different metabolic substrates. The metabolic conversion of various substrates was measured according to the manufacturer’s recommendations using a kinetic multimode plate reader (Tecan) at *λ*_ex_ 535 nm and *λ*_em_ 590 nm set to 37 °C for 4 h. The values were normalised to the control cells, and fold changes were calculated.

### Quantitative real-time PCR

For gene expression analysis, total mRNA from tumour cells were isolated (Bio&Sell, Feucht, Germany) 6 h post plasma treatment. By employing the PrimeScript cDNA synthesis kit (Takara Bio, Kusatsu, Japan), 500 ng of mRNA was converted to cDNA. β-actin was used as an internal control, and the expression of genes was evaluated according to the primer sequences listed (Table 1). All validated primers were purchased from Sigma–Aldrich. qPCR assays were carried out using TB Green Premix Ex Taq II (Tli RNase H Plus) reagent (Takara Bio). The QuantStudio 1 system (Thermo Fisher Scientific) was set to the following cycling parameters: 95 °C denaturation for 30 s; 40 cycles of 95 °C for 5 s followed by 60 °C for 30 s; 95 °C for 5 s; 60 °C for 1 min; followed by a dissociation step. The results were expressed as fold change and calculated using the _ΔΔ_Ct method relative to a control sample. Data analysis was done using the QuantStudio Design and Analysis software (Thermo Fisher Scientific)

**Table 1:** List of primers used in the current study.

S.no	Gene	Primer pairs	Function
1	ASCT2	Fwd: GAGCTGCTTATCCGCTTCTTC Rev: GGGGCGTACCACATGATCC	Amino acid transport
2	BCL2	Fwd: GTCTTCGCTGCGGAGATCAT Rev: CATTCCGATATACGCTGGGAC	Apoptosis
3	BNIP3	Fwd: ATGTCGTCCCACCTAGTCGAG Rev: TGAGGATGGTACGTGTTCCAG	Apoptosis
4	EGLN^a^	Fwd: TCGCAACCCTCATGAAGTACAA Rev: ACTTTAGCTCGTGCTCTCTCA	Oxygen response
5	FABP3	Fwd: GGCACCTGGAAGCTAGTGG Rev: CTGCCTGGTAGCAAAACCC	Fatty acid transport
6	FABP7^a^	Fwd: CCACTGCAGATGATAGAAAC Rev: TCATAACCATTTTGCCATCC	Fatty acid transport
7	G6PD	Fwd: CGAGGCCGTCACCAAGAAC Rev: GTAGTGGTCGATGCGGTAGA	Pentose Phosphate Pathway
8	GAPDH	Fwd: ACAGTTGCCATGTAGACC Rev: TTGAGCACAGGGTACTTTA	Glycolysis
9	GLS	Fwd: GGAGCCTCGTCTCGATGCTA Rev: GAGAGAAGATGCGTCCGGT	Glutamine metabolism
10	GLUT1	Fwd: GCCAGAAGGAGTCAGGTTCAA Rev: TCCTCGGAAAGGAGTTAGATCC	Glucose import
11	HEY1	Fwd: GTTCGGCTCTAGGTTCCATGT Rev: CGTCGGCGCTTCTCAATTATTC	Notch signaling
12	HIF-1α	Fwd: TTCCCGACTAGGCCCATTC Rev: CAGGTATTCAAGGTCCCATTTCA	Oxygen response
13	HK1	Fwd: ATCACGGATGTATGACGTTTTGG Rev: CAGGCTATTGCTGCGAAGAAC	Glycolysis
14	LAT1	Fwd: CACCCTACCTGTGGCATTATTG Rev: CCATTCTTGTCCGATGGTTCAT	Amino acid transport
15	LAT2^a^	Fwd: AACGCGAGTGACCAGAAAGT Rev: GAAGCCCGTCACAATCCAGA	Amino acid transport
16	LPIN	Fwd: CCAGCCCAATGGAAACCTCC Rev: AGGTGCATAGGGATAACTTCCTG	Phospholipid synthesis
17	MCT1	Fwd: GTGACTGCAGGGTTCGAGTA Rev: CTGCTGATAGGACCTCCACC	Monocarboxylate transport
18	MCT2	Fwd: GGGTTGGATTGTGGTTGGAG Rev: TCCTGCGTACATAACAGCCAG	Monocarboxylate transport
19	MPC1	Fwd: ATGTTCGAGTTCCTCAGC Rev: ACTTCACCTTGATGGTCTC	Pyruvate transport
20	OGDH	Fwd: CATCGACAAATCCAGCGAGA Rev: ATCCTCTCATGGTACATGCCC	TCA
21	OXCT1	Fwd: GTTGGTGGTTTTGGGCTATGT Rev: AGACCATGCGTTTTATCTGCTT	TCA
22	PKM2	Fwd: CATGGCTCCTACGGAGAGGT Rev: ACATGGAACGCTTTACCGCAT	Glycolysis
23	PLIN2	Fwd: TCTGGCTAGTGCTCAAGAAGA Rev: TAACCCACGACTCCCATCTCG	Fatty acid metabolism
24	SIAH2	Fwd: TCTTCGAGTGTCCGGTCTG Rev: CGGCATTGGTTACACACCAG	Ubiquitination
25	SIRT-4	Fwd: GCTTTGCGTTGACTTTCAGGT Rev: CCAATGGAGGCTTTCGAGCA	Metabolic regulation
26	SLC16A3	Fwd: CCATGCTCTACGGGACAGG Rev: GCTTGCTGAAGTAGCGGTT	Fatty acid metabolism
27	SLC22A16	Fwd: GGAATAAGAGGGAGAACACATCG Rev: TCACCGCAGTGCTTTTCCA	Fatty acid metabolism
28	SLC25A10	Fwd: ACCTGCTCAAGGTGCATCTG Rev: CAGGGAGTAGGTCATCTGTCTG	TCA
29	SLC25A20	Fwd: GACCAGCCAAAACCCATCAG Rev: AGAGGGTGACCGACGAACA	Fatty acid metabolism
30	SLC3A2	Fwd: TGAATGAGTTAGAGCCCGAGA Rev: GTCTTCCGCCACCTTGATCTT	Amino acid transport
31	SOX9	Fwd: AGCGAACGCACATCAAGAC Rev: CTGTAGGCGATCTGTTGGGG	Stem cell development

^a^Genes not detected in the cell lines tested

### esiRNA knockdown

siRNA-mediated knockdown experiments were carried out using an endoribonuclease-prepared siRNA (esiRNA) pool consisting of mixtures of different siRNAs that share the same on-target sequence but differ in their sequence-dependent off-target signatures. Cells (1 × 10^4^) were plated in 96-well plates. Sixteen hours later, cells were transfected with esiRNA targeting ASCT2 mRNA (Sigma–Aldrich, Darmstadt, Germany) using RNAiMAX (Thermo Fisher Scientific) according to the manufacturer’s recommendation. Twenty-four hours later, the medium was exchanged with RPMI medium supplemented with 2% FBS. Cells were then exposed to plasma for 60 s, and viability and metabolic activity was measured after 6 h. Representative protein lysates were used to determine the knockdown efficiency of ASCT2 protein by immunoblotting.

### Immunoblotting

Cells were harvested in ice-cold PBS and lysed in RIPA buffer (Thermo Fisher Scientific) supplemented with complete protease and phosphatase inhibitors (PIM complete; Roche, Mannheim, Germany) for 20 min on ice. After centrifugation at 15,000 × *g* for 15 min at 4 °C, the cell extracts’ total protein content was quantified using Rotiquant (Carl Roth, Karlsruhe, Germany). Forty micrograms of protein were resolved by SDS-PAGE (Invitrogen) and blotted on PVDF membranes (Invitrogen). The membranes were probed with anti-ASCT2, anti-β actin, and anti-LAT1 primary antibodies (Cell Signaling Technology, Danvers, Massachusetts, USA) followed by incubation with secondary horseradish peroxidase-coupled antibodies (Santa Cruz Biotechnology, Dallas, Texas, USA). Signals were acquired using a chemiluminescence detection system (Applied Biosystems, Foster City, California, USA) in the linear dynamic range.

### Amino acid supplementation assay

Cells (1 × 10^4^/well) were plated in 96-well plates. Sixteen hours later, the medium was exchanged with RPMI medium supplemented with 2% FBS. Cells were exposed to plasma for 60 s. Six hours later, the medium was replaced with an amino acid-free medium (EBSS with 2% FBS). Basal metabolic activity (resazurin; 50 µM) was measured for 90 min (at 30 min intervals). At the 90th min, selected wells were supplemented with glutamine, valine, or tyrosine (each at a final concentration of 200 µM), and the metabolic activity was measured for a total of 4 h. The extent of the amino acid supplementation affecting the cellular response was quantified by calculating the area under the curve obtained for the metabolic activity. For plasma treatment, normalised results were displayed as percentage of non-supplemented cells exposed to plasma.

### Statistical analysis

Graphing, including heatmaps and volcano plots, and statistical analysis was done using *prism* 8.4.3 (GraphPad Software, USA). The number of experiments and type of statistical analysis is given in the figure legends and was *t*-test when comparing two groups, and analysis of variances (anova) when using multiple groups. For differentially gene expression analysis, prism-embedded multiple *t*-tests were used. Genes with a fold change of ≥2, and a *p*-value < 0.05 were considered to be significant. Level of significance was indicated as follows: α = 0.05 (*), α = 0.01 (**), α = 0.001 (***).

## Results

### Exposure to plasma-induced distinct metabolic profiles in human tumour cells

The consumption of metabolic substrates in four human tumour cell lines (Panc-1, HeLa, SK-MEL-28 and OVCAR3) was assessed 1 h after plasma treatment using the MitoPlate mitochondria function assay (Fig. 1a). This assay is composed of 31 substrates involved in multiple metabolic pathways. The changes in substrate consumption are represented in heatmaps (Fig. 1b) of ketones/fatty acids, glycolysis, amino acids and tricarboxylic acid (TCA) pathways, and were normalised to the consumption of each of the untreated cell line. Panc-1 and HeLa cells showed increased differential consumption of metabolic substrates of individual pathways compared to OVCAR3 and SK-MEL-28 cells (Fig. 1c). Furthermore, both Panc-1 and HeLa cells displayed increased overall consumption across all substrates following plasma treatment (Fig. S1A). The resazurin-based quantification of metabolic activity 24 h after plasma treatment revealed that Panc-1 and HeLa had a higher survival fraction compared to SK-MEL-28 and OVCAR3 cells (Fig. S1B). These data indicated that the ability of tumour cells to utilise specific substrates might contribute to extended survival following plasma treatment. The above findings also confirmed that individual tumour cell types respond by activation of multiple metabolic pathways following plasma exposure.

### Plasma-treated tumour cells differentially regulated genes of metabolic pathways

**Fig. 2:**
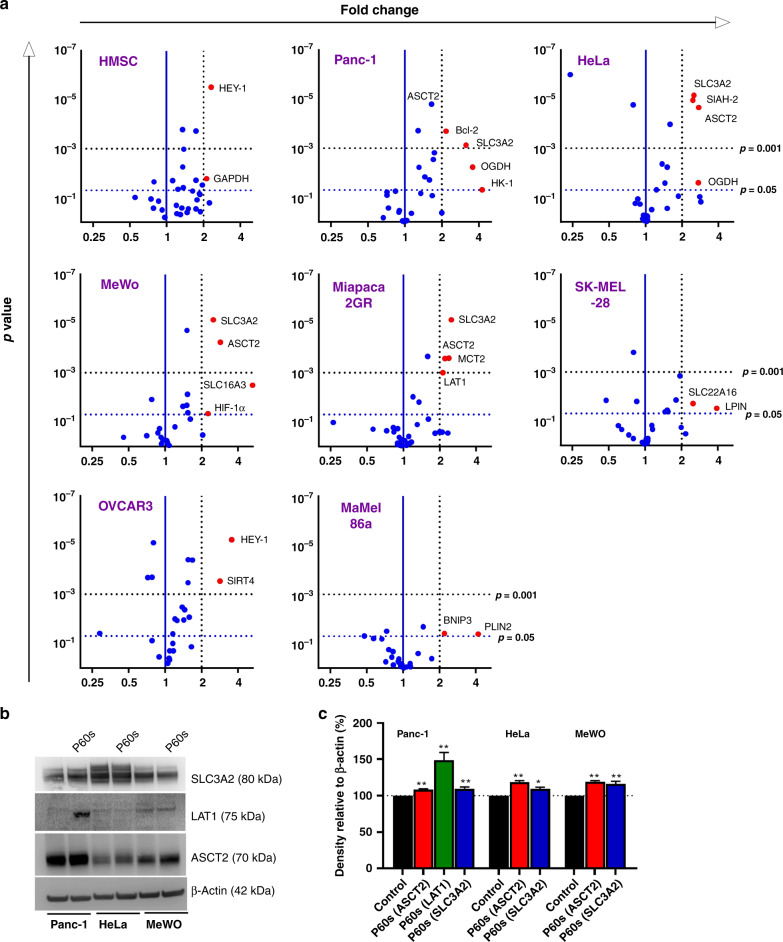
Differentially expressed metabolic genes following plasma exposure in tumour cells. **a** volcano plots indicating the differentially expressed genes (red) in HMSC, Panc-1, HeLa, MeWo, Miapaca2GR, SK-MEL-28, OVCAR3 and MaMel86a cell lines 6 h post gas plasma treatment with the vertical blue line in the middle indicating a fold change in gene expression of 1, the dotted vertical line indicating the threshold of a two-fold change in gene expression, and the horizontal dotted lines indicating a threshold of *p* = 0.05 and 0.001, respectively; **b** immunoblotting confirming the expression of SLC3A2, LAT1 and ASCT2 in Panc-1, HeLa and MeWo cell lines 6 h post plasma exposure. Data are mean derived from two to three independent experiments (B = n1).

Since there was a utilisation of substrates from multiple metabolic pathways following plasma treatment, our goal was to identify the key genes/pathways that were differentially induced following exposure to plasma. Seven human tumour cell lines and one non-tumorigenic cell line (HMSC) were treated with plasma for 60 s (P60s), and total RNA was isolated 6 h later. The expression of 32 genes encompassing the metabolic pathways was assessed using qPCR (Table 1). Differentially expressed genes from each cell line are depicted in (Fig. 2a). The expression of MCT2, LAT1, SLC16A3, BCL2, OGDH, HK1, SIAH2 and SLC3A2 was upregulated (>2-fold change) in, e.g. HeLa, Panc-1, MeWo and Miapaca2GR cells but not in HMSC, SK-MEL-28, Mamel86a and OVCAR3 cells (Table 2). Among those targets, only ASCT2 and SLC3A2 were consistently upregulated across the Panc-1 and HeLa cell lines that were less affected by the plasma treatment (Fig. S1B). Western blotting (Fig. 2b) and densitometric analysis (Fig. 2c) suggested a marginal increase of SLC3A2 and ASCT2 in HeLa, Panc-1 and MeWo cells following plasma treatment. Panc-1 cells showed an increase in LAT1 (~75 kDa) on the protein level.

**Table 2:** Genes differentially upregulated following plasma exposure.

Gene	Function	Pathway
ASCT2	Transporter	Amino acid
BCL2	Anti-apoptotic	Apoptosis
HIF1A	Transcription factor	Oxygen response
HK1	Enzymatic	Glycolysis
LAT1	Transporter	Amino acid
OGDH	Enzymatic	TCA
SIAH2	Enzymatic	Ubiquitination
SLC16A3	Transporter	Glycolysis
SLC3A2	Co-activator	Amino acid

### ASCT2-mediated glutamine metabolism was essential for tumour cell survival following plasma treatment

**Fig. 3:**
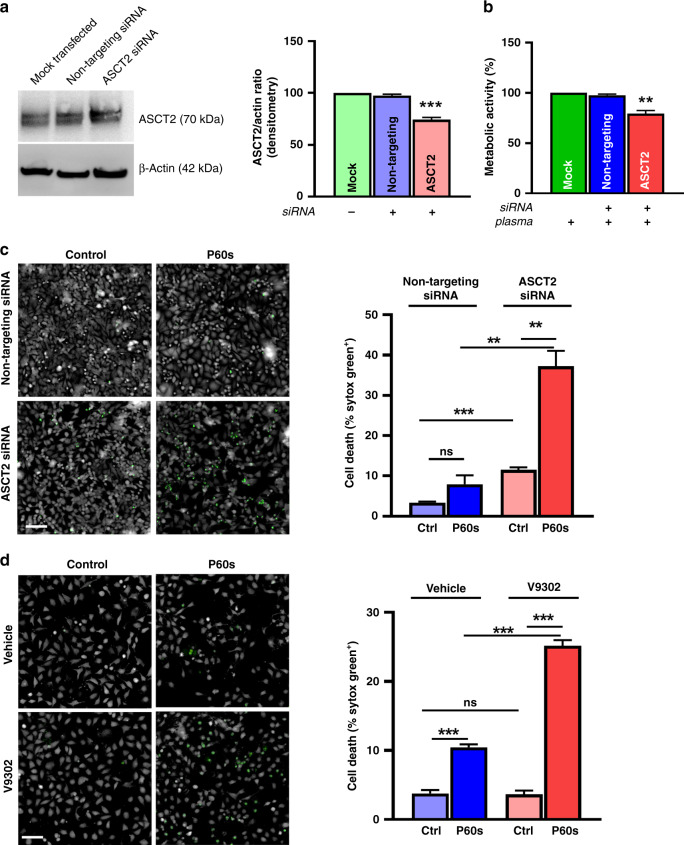
ASCT2 inhibition sensitised HeLa cells to plasma treatment. **a** immunoblotting and densitometry of ASCT2 knockdown in the HeLa cell line; **b, c** metabolic activity (**b**) and viability (**c**) of ASCT2 knockdown in HeLa cells 6 h after plasma exposure; **d** viability of HeLa cells following pre-treatment with the ASCT2 inhibitor V9302 (10 µM), followed by 6 h plasma treatment. Data are mean ± SEM derived from three independent experiments with *p < 0.05, **p < 0.01 and ***p < 0.001. Statistical analysis was done using one-way ANOVA with Tukey post-test for multiple comparisons, or two-tailed *t*-test. Scale bar is 100 μm.

ASCT2 catalyses the exchange of neutral amino acids, including glutamine, as an alternative carbon source for the TCA cycle. To implicate the role of ASCT2 in extended survival of tumour cells, we performed siRNA-mediated knockdown of ASCT2 in HeLa cells. Quantification of immunoblots (Fig. 3a) revealed a 25% knockdown of ASCT2 at the protein level. These cells were then treated with plasma, and metabolic activity (Fig. 3b) and viability (Fig. 3c) assays were performed. ASCT2 knockdown led to decreased metabolic activity and increased cytotoxicity 24 h post plasma treatment. Furthermore, pre-treatment of tumour cells with V9302, a small molecule inhibitor against ASCT2, sensitised HeLa cells to plasma treatment (Fig. 3d). Along similar lines, glutamine-deprived culture media sensitised both MeWo (Fig. S2A) and Panc-1 (Fig. S2B) cells to plasma treatment. These results indicated that some tumour cell lines depended on ASCT2 expression and glutamine metabolism to protect themselves from the toxic consequences of plasma-derived ROS.

### Amino acid supplementation rescued tumour cells from plasma insult

**Fig. 4:**
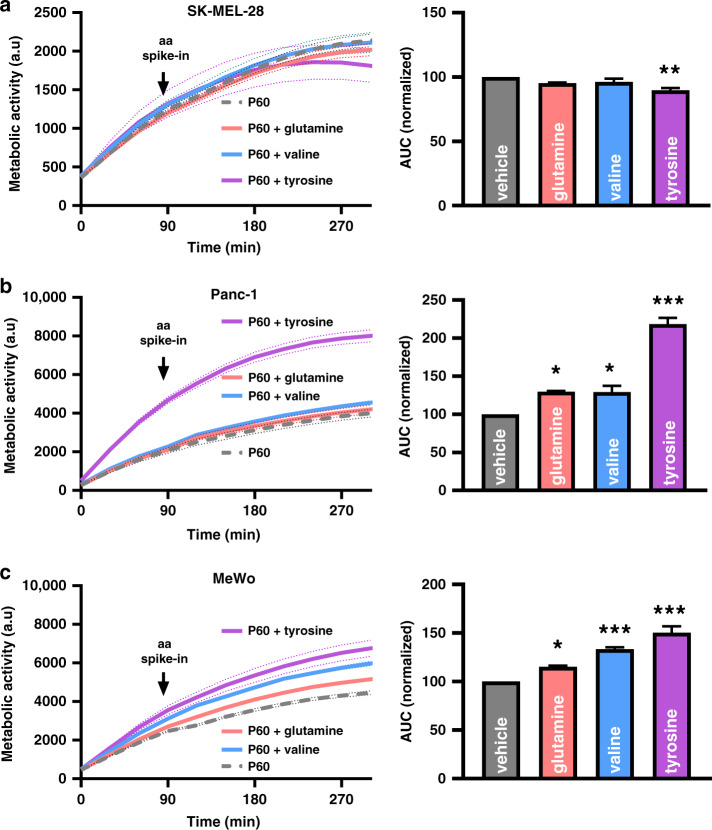
Metabolic activity of tumour cells following exogenous amino acid supplementation. Kinetic of metabolic activity and normalised area under the curve (AUC) of plasma-treated SK-MEL-28 (**a**), Panc-1 (**b**), and MeWO (**c**) cells with or without supplementation of exogenous glutamine, valine or tyrosine (200 μM). Data are mean ± SEM derived from three independent experiments. *p < 0.05; **p < 0.01; ***p < 0.001. Statistical analysis was done using one-way ANOVA with Tukey post-test for multiple comparisons. aa amino acid.

**Fig. 5:**
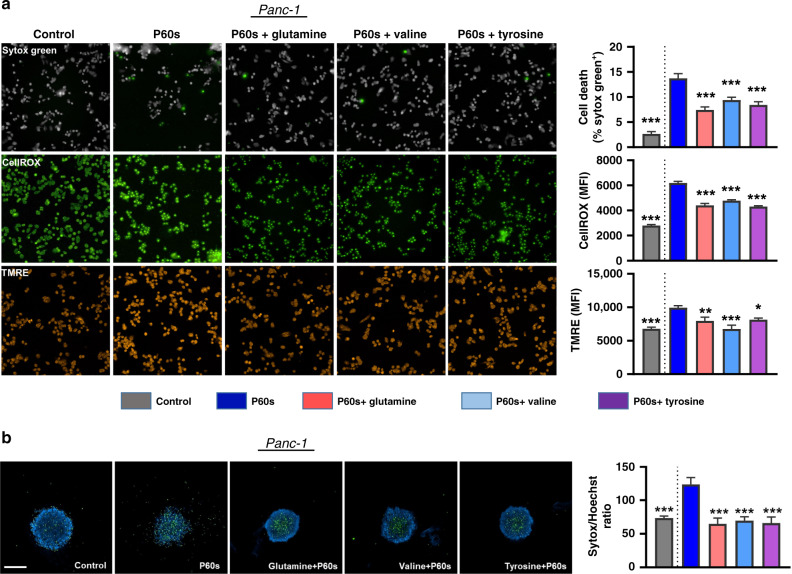
Exogenous amino acid supplementation inhibits plasma-mediated oxidative stress and viability. **a** representative images and quantification of viability (sytox green), cellular ROS (CellROX green) and mitochondrial membrane potential (TMRE) of plasma-treated Panc-1 cells supplemented with exogenous glutamine, valine or tyrosine (200 μM). Scale bar is 100 μm. **b** representative images and quantification of viability (sytox green) in Panc-1 derived spheroids supplemented with exogenous glutamine, valine or tyrosine (200 μM). Scale bar is 500 μm. Data are mean ± SEM derived from three independent experiments with *p < 0.05, **p < 0.01 and ***p < 0.001. Statistical analysis was done using one-way ANOVA with Tukey post-test for multiple comparisons.

ASCT2 and SLC3A2 are localised in the plasma membrane and transport various amino acids from the extracellular environment. To validate exogenous amino acids’ role in the pro-survival phenotype, amino acid pulse assays were performed. Representative cell lines were treated with plasma and incubated in amino acid-free media for the following 3 h. Basal metabolic activity was measured for 90 min, followed by 200 μM glutamine, valine or tyrosine. The kinetic measurements were then continued for another 3 h. SK-MEL-28 cells did not show any influence in the metabolic activity after the addition of glutamine or valine but a notable reduction upon tyrosine supplementation (Fig. 4a), whereas Panc-1 (Fig. 4b) and MeWo cells (Fig. 4c) showed an increase in metabolic activity following the amino acid pulse. This assay’s specificity was demonstrated by the increased metabolic activity observed following the addition of the LAT1-specific amino acid tyrosine in Panc-1 cells (Fig. 2b vs. Fig. 4b). Untreated tumour cells showed only a modest increase in metabolic activity following amino acid pulsing due to the transporters’ basal expression levels (Fig. S3). These results confirmed that specific tumour cell types rely on exogenous amino acids to enhance their metabolic function following plasma-induced ROS treatment, likely for coping with the resultant oxidative stress. To confirm the downstream consequence of the metabolic activity changes, the mitochondrial membrane potential, ROS levels and cell viability 6 h post amino acid pulse were investigated using quantitative high content imaging. Panc-1 cells showed reduced oxidative stress and cytotoxicity following amino acid supplementation (Fig. 5a). The integrity of the mitochondrial membrane potential was sustained. However, this effect was not seen in SK-MEL-28 cells (Fig. S4A), correlating with the findings above (Fig. 4a). The exogenous amino acid supplementation also rescued Panc-1 spheroids in 3D culture 72 h post-treatment **(**Fig. 5b). These results suggest that a subset of tumour cells activates the expression of genes involved in amino acid transport, thereby leading to a pro-survival phenotype following plasma exposure to attenuate ROS-mediated cytotoxicity.

## Discussion

Cold physical plasma is an evolving technology employed to target tumour cells by delivering ROS directly to the tumour tissue.^24^ Since tumour cells are vulnerable to exogenous ROS, physical plasma treatment modalities provide a distinct advantage in the selective elimination of tumour cells.^25^ However, the ability of ROS to initiate a variety of signalling mechanisms^26^ and the observed tumour heterogeneity^27^ necessitates a more detailed understanding of how tumour cells respond to plasma.

Although glucose is the principal source of energy in cells and anti-glycolytic agents augment toxic plasma effects,^28^ amino acids are an important class of nutrients obligatory for cell survival. While some amino acids have specific biologic functions in metabolic processes, epigenetic regulation, and GSH synthesis,^20^ the primary physiologic function is to serve as building blocks for protein synthesis.^29^ Tumour cells employ opportunistic strategies to obtain these metabolic substrates during the exponential growth phase or to bypass therapeutic interventions. Since ROS induce gene expression and modulate protein activity, we employed a two-step screening approach to understand the metabolic signatures in tumour cell lines. The substrate utilisation in tumour cells assessed 1-h post plasma treatment could be attributed to the early post-translational modifications (e.g. phosphorylation, s-glutathionylation and nitration) of various enzymes and proteins of the TCA, glycolysis fatty acid and amino acid pathways.^30^ However, the mRNA expression analysis was carried out at 6 h post plasma exposure, to include specific gene expression patterns induced by redox-regulated transcription factors.^19^ Examining these datasets from several tumour cell lines revealed amino acid substrates, and its corresponding membrane transporters play a central role in metabolic reprogramming in tumour cells.

In our study, ASCT2 and SLC3A2 were consistently upregulated in tumour cell lines that exhibited prolonged survival following plasma treatment. The importance of these genes was further validated using a non-transformed HSMC cell line. ASCT2 belongs to the group of system ASC, transporting alanine, glutamine, serine and cysteine along with other aliphatic amino acids.^31^ SLC3A2 is an essential transmembrane protein critical for activating LAT1/2/3, ASC1, BAT1 and xCT involved in amino acid transport.^32–34^ Supplementation of exogenous ASCT2-specific amino acids (glutamine and valine) led to improved metabolic activity and survival in plasma-treated tumour cells in Panc-1 and MeWo but not in SK-MEL-28 cells. Glutamine is the primary amino acid that drives the TCA cycle and sustains ATP production.^35^ In the absence of glucose, glutamine-derived fumarate malate and citrate are increased in a glucose independent TCA cycle.^36^ Vice versa, the pharmacological or genetic inhibition of ASCT2 sensitised the tumour cells to plasma-induced cell death. These results validate that tumour cells are dependent on extracellular substrates for their biosynthetic machinery during stress by activation of respective transporters.

Interestingly, the LAT1-specific amino acid tyrosine also improved the metabolic activity in Panc-1 cells. The qPCR screen did not detect significant changes in LAT1 expression in Panc-1 cells, but there was a significant induction at the protein level following plasma exposure. This could be due to an observed alternate LAT1 isoform (~70 kDa) induced in these cells leading to tyrosine uptake. The increase in metabolic function due to amino acid supplementation also reduced oxidative stress and partially restored mitochondrial membrane potential and viability in Panc-1 cells. However, the comparable effect was not observed in SK-MEL-28 cells as it lacked the induction of transporter expression following plasma exposure. Our screen also identified the consumption of fatty acid and TCA substrates. It has been previously described that amino acids can supply carbon atoms for lipid biosynthesis and the TCA cycle via sustained acetyl-CoA pools.^29,37^ The maintenance of cellular amino acid pools is also regulated by autophagy, evoked during cellular stress, thereby providing substrates for energy production in tumour cells.^38,39^ It could be speculated from the current study that uptake of exogenous amino acids could be the first choice to maintain cellular amino acid pools, after which cell may commit to autophagy. The observed metabolic alterations in our study could also have a direct consequence in the tumour microenvironment (TME). Extracellular glutamine supports the differentiation of T cells into TH_1_ T cells.^40^ Targeting glutamine metabolism in tumour cells by CB-839/anti-PD-1/PD-L1 led to the depletion of glutamine in the tumour microenvironment (TME), enabling potent anti-tumour immune responses.^41^ However, further studies are warranted to differentiate amino acid pools derived from exogenous and de novo sources following plasma treatment and their influence in the TME. A previous study highlighted the importance of D-glutamine and D-glutamate metabolism in leukemic cells exposed to cold plasma.^42^ Investigating plasma-treated myeloma cells, the authors demonstrated in another study a strong enrichment of the β-alanine, propanoate, and linoleic acid metabolism, while the metabolic pathways for alanine, aspartate, glutamate, arginine, and proline were found to be enriched as well, albeit to a lesser extent.^43^ Our previous study determined that the cystine/glutamate antiporter xCT is induced in tumour cells following plasma exposure, leading to treatment resistance.^18^ In the current study, we implicate exogenous amino acids in the prolonged survival of some tumour cells. However, the involvement of other amino acid transporters (not included in this study) cannot be ruled out. Apart from this, another recent study suggested the involvement of purine metabolism and Pantothenate and CoA biosynthesis in both non-malignant and malignant cells following plasma exposure in vitro.^44^

Taken together, there is strong evidence that amino acids play a vital role in metabolic reprogramming in tumour cells. Due to multiple substrate specificities of the amino acid transporters,^31^ and rampant dosage compensation in the SLC gene family,^45,46^ single-transport inhibitors are unlikely to be effective against cancer therapy. Hence, combination with plasma and specific transport inhibitors could be useful in the targeting of tumour cells.

## Supplementary information

Supplemental material

## Data Availability

The datasets used and/or analysed during the current study are available from the corresponding author on reasonable request.

## References

[1] Privat-Maldonado A, Schmidt A, Lin A, Weltmann KD, Wende K, Bogaerts A (2019). ROS from physical plasmas: redox chemistry for biomedical therapy. Oxid. Med. Cell Longev..

[2] Graves DB (2017). Mechanisms of plasma medicine: coupling plasma physics, biochemistry, and biology. IEEE Trans. Radiat. Plasma Med. Sci..

[3] Mizuno K, Shirakawa Y, Sakamoto T, Ishizaki H, Nishijima Y, Ono R (2018). Plasma-induced suppression of recurrent and reinoculated melanoma tumors in mice. IEEE TRPMS.

[4] Bekeschus S, Clemen R, Niessner F, Sagwal SK, Freund E, Schmidt A (2020). Medical gas plasma jet technology targets murine melanoma in an immunogenic fashion. Adv. Sci..

[5] Lin A, Gorbanev Y, De Backer J, Van Loenhout J, Van Boxem W, Lemiere F (2019). Non-thermal plasma as a unique delivery system of short-lived reactive oxygen and nitrogen species for immunogenic cell death in melanoma cells. Adv. Sci..

[6] Brullé, L., Vandamme, M., Riès, D., Martel, E., Robert, E., Lerondel, S. et al. Effects of a Non Thermal Plasma Treatment Alone or in Combination with Gemcitabine in a MIA PaCa2-luc Orthotopic Pancreatic Carcinoma Model. *PLoS ONE* **7**, e52653. 10.1371/journal.pone.0052653 (2012). 10.1371/journal.pone.0052653 PMC3530450 23300736

[7] Liedtke KR, Bekeschus S, Kaeding A, Hackbarth C, Kuehn JP, Heidecke CD (2017). Non-thermal plasma-treated solution demonstrates antitumor activity against pancreatic cancer cells in vitro and in vivo. Sci. Rep..

[8] Hattori N, Yamada S, Torii K, Takeda S, Nakamura K, Tanaka H (2015). Effectiveness of plasma treatment on pancreatic cancer cells. Int. J. Oncol..

[9] Freund E, Liedtke KR, van der Linde J, Metelmann HR, Heidecke CD, Partecke LI (2019). Physical plasma-treated saline promotes an immunogenic phenotype in CT26 colon cancer cells in vitro and in vivo. Sci. Rep..

[10] Lin AG, Xiang B, Merlino DJ, Baybutt TR, Sahu J, Fridman A (2018). Non-thermal plasma induces immunogenic cell death in vivo in murine CT26 colorectal tumors. Oncoimmunology.

[11] Sagwal SK, Pasqual-Melo G, Bodnar Y, Gandhirajan RK, Bekeschus S (2018). Combination of chemotherapy and physical plasma elicits melanoma cell death via upregulation of SLC22A16. Cell Death Dis..

[12] Wang K, Jiang J, Lei Y, Zhou S, Wei Y, Huang C (2019). Targeting metabolic-redox circuits for cancer therapy. Trends Biochem. Sci..

[13] Panieri E, Santoro MM (2016). ROS homeostasis and metabolism: a dangerous liason in cancer cells. Cell Death Dis..

[14] Diebold, L. & Chandel, N. S. Mitochondrial ROS regulation of proliferating cells. *Free Radic. Biol. Med.* **100**, 86–93 (2016). 10.1016/j.freeradbiomed.2016.04.198 27154978

[15] Sabharwal SS, Schumacker PT (2014). Mitochondrial ROS in cancer: initiators, amplifiers or an Achilles’ heel?. Nat. Rev. Cancer.

[16] Bansal A, Simon MC (2018). Glutathione metabolism in cancer progression and treatment resistance. J. Cell Biol..

[17] Hill BG, Bhatnagar A (2012). Protein S-glutathiolation: redox-sensitive regulation of protein function. J. Mol. Cell Cardiol..

[18] Bekeschus S, Eisenmann S, Sagwal SK, Bodnar Y, Moritz J, Poschkamp B (2020). xCT (SLC7A11) expression confers intrinsic resistance to physical plasma treatment in tumor cells. Redox Biol..

[19] Ye P, Mimura J, Okada T, Sato H, Liu T, Maruyama A (2014). Nrf2- and ATF4-dependent upregulation of xCT modulates the sensitivity of T24 bladder carcinoma cells to proteasome inhibition. Mol. Cell Biol..

[20] Lieu EL, Nguyen T, Rhyne S, Kim J (2020). Amino acids in cancer. Exp. Mol. Med..

[21] Bekeschus S, Schmidt A, Niessner F, Gerling T, Weltmann KD, Wende K (2017). Basic research in plasma medicine—a throughput approach from liquids to cells. J. Vis. Exp..

[22] Reuter, S., von Woedtke, T. & Weltmann, K. D. The kINPen-a review on physics and chemistry of the atmospheric pressure plasma jet and its applications. *J. Phys. D Appl. Phys*. **51**, 233001–52 (2018).

[23] Gandhirajan RK, Rodder K, Bodnar Y, Pasqual-Melo G, Emmert S, Griguer CE (2018). Cytochrome C oxidase inhibition and cold plasma-derived oxidants synergize in melanoma cell death induction. Sci. Rep..

[24] Hirst AM, Frame FM, Arya M, Maitland NJ, O’Connell D (2016). Low temperature plasmas as emerging cancer therapeutics: the state of play and thoughts for the future. Tumour Biol..

[25] Liedtke KR, Freund E, Hermes M, Oswald S, Heidecke CD, Partecke LI (2020). Gas plasma-conditioned Ringer’s lactate enhances the cytotoxic activity of cisplatin and gemcitabine in pancreatic cancer in vitro and in ovo. Cancers.

[26] Moloney JN, Cotter TG (2018). ROS signalling in the biology of cancer. Semin. Cell Dev. Biol..

[27] Grzywa TM, Paskal W, Wlodarski PK (2017). Intratumor and intertumor heterogeneity in melanoma. Transl. Oncol..

[28] Kaushik N, Lee SJ, Choi TG, Baik KY, Uhm HS, Kim CH (2015). Non-thermal plasma with 2-deoxy-D-glucose synergistically induces cell death by targeting glycolysis in blood cancer cells. Sci. Rep..

[29] Yoneshiro T, Wang Q, Tajima K, Matsushita M, Maki H, Igarashi K (2019). BCAA catabolism in brown fat controls energy homeostasis through SLC25A44. Nature.

[30] Wang K, Jiang JW, Lei YL, Zhou ST, Wei YQ, Huang CH (2019). Targeting metabolic-redox circuits for cancer therapy. Trends Biochem. Sci..

[31] Fuchs BC, Bode BP (2005). Amino acid transporters ASCT2 and LAT1 in cancer: partners in crime?. Semin. Cancer Biol..

[32] Sato H, Nomura S, Maebara K, Sato K, Tamba M, Bannai S (2004). Transcriptional control of cystine/glutamate transporter gene by amino acid deprivation. Biochem. Biophys. Res. Commun..

[33] Napolitano L, Scalise M, Galluccio M, Pochini L, Albanese LM, Indiveri C (2015). LAT1 is the transport competent unit of the LAT1/CD98 heterodimeric amino acid transporter. Int. J. Biochem. Cell Biol..

[34] Cano-Crespo, S., Chillaron, J., Junza, A., Fernandez-Miranda, G., Garcia, J., Polte, C. et al. CD98hc (SLC3A2) sustains amino acid and nucleotide availability for cell cycle progression. *Sci. Rep*. **9**, 14065–84 (2019). 10.1038/s41598-019-50547-9 PMC6773781 31575908

[35] Hensley CT, Wasti AT, DeBerardinis RJ (2013). Glutamine and cancer: cell biology, physiology, and clinical opportunities. J. Clin. Invest..

[36] Le A, Lane AN, Hamaker M, Bose S, Gouw A, Barbi J (2012). Glucose-independent glutamine metabolism via TCA cycling for proliferation and survival in B cells. Cell Metab..

[37] Metallo CM, Gameiro PA, Bell EL, Mattaini KR, Yang J, Hiller K (2011). Reductive glutamine metabolism by IDH1 mediates lipogenesis under hypoxia. Nature.

[38] Guo JY, Teng X, Laddha SV, Ma SR, Van Nostrand SC, Yang Y (2016). Autophagy provides metabolic substrates to maintain energy charge and nucleotide pools in Ras-driven lung cancer cells. Genes Dev..

[39] Kuma A, Mizushima N (2010). Physiological role of autophagy as an intracellular recycling system: With an emphasis on nutrient metabolism. Semin. Cell Dev. Biol..

[40] Klysz, D., Tai, X. G., Robert, P. A., Craveiro, M., Cretenet, G., Oburoglu, L. et al. Glutamine-dependent alpha-ketoglutarate production regulates the balance between T helper 1 cell and regulatory T cell generation. *Sci. Signal.* **8**, ra97 (2015). 10.1126/scisignal.aab2610 26420908

[41] Gross, M., Chen, J., Engler, J., Janes, J., Leone, R., MacKinnon, A. et al. Glutaminase inhibition with CB-839 enhances anti-tumor activity of PD-1 and PD-L1 antibodies by overcoming a metabolic checkpoint blocking T cell activation. *Cancer Res*. **76**, (2016).

[42] Xu D, Ning N, Xu Y, Wang B, Cui Q, Liu Z (2019). Effect of cold atmospheric plasma treatment on the metabolites of human leukemia cells. Cancer Cell Int..

[43] Xu D, Xu Y, Ning N, Cui Q, Liu Z, Wang X (2018). Alteration of metabolite profiling by cold atmospheric plasma treatment in human myeloma cells. Cancer Cell Int..

[44] Tian, M., Xu, D., Li, B., Wang, S., Qi, M., Zhang, H. et al. Metabolome analysis of selective inactivation of human melanoma and normal cells by cold atmospheric plasma. *Plasma Chem. Plasma Process*. 591–605 (2021).

[45] Cutler MJ, Choo EF (2011). Overview of SLC22A and SLCO families of drug uptake transporters in the context of cancer treatments. Curr. Drug Metab..

[46] Broer A, Gauthier-Coles G, Rahimi F, van Geldermalsen M, Dorsch D, Wegener A (2019). Ablation of the ASCT2 (SLC1 A5) gene encoding a neutral amino acid transporter reveals transporter plasticity and redundancy in cancer cells. J. Biol. Chem..

