# Supplementary material for: The amino acid metabolism is essential for evading physical plasma-induced tumour cell death
Source: Br J Cancer. 2021 Mar 25;124(11):1854–63. doi: 10.1038/s41416-021-01335-8 (PMC8144554; doi:10.1038/s41416-021-01335-8)

# Supplementary figure legends

**Figure S1.** (A) total substrate consumption (%) of the indicated cell lines from the MitoPlate assay; (B) metabolic activity of tumor cells 6h post plasma treatment. Data are mean ± SEM derived from three independent experiments with * *p <* 0.05, ** *p <* 0.01, and *** *p <* 0.001. Statistical analysis was done using one-way ANOVA with Tukey post-test for multiple comparisons. Scale bar is 100 μm.

**Figure S2.** (A-B) sytox green viability staining of MeWo (A) and Panc-1 (B) cells 6h following plasma treatment in the presence or absence of glutamine. Data are mean ± SEM derived from three independent experiments with * *p <* 0.05, ** *p <* 0.01, and *** *p <* 0.001. Statistical analysis was done using one-way ANOVA with Tukey post-test for multiple comparisons. Scale bar is 100 μm.

**Figure S3.** Kinetic of metabolic activity and normalized area under the curve (AUC) of untreated SK-MEL-28 (A), Panc-1 (B), and MeWO (C) cells with or without supplementation of exogenous glutamine, valine, or tyrosine (200 μM). Data are mean ± SEM derived from three independent experiments. * *p <* 0.05; ** *p <* 0.01. Statistical analysis was done using one-way ANOVA with Tukey post-test for multiple comparisons, or two-tailed t-test.

**Figure S4.** **Exogenous amino acid supplementation does not inhibit plasma-mediated oxidative stress and viability** **in SK-MEL-28 cells**. (A) representative images and quantification of viability (sytox green), cellular ROS (CellROX green), and mitochondrial membrane potential (TMRE) of plasma-treated SK-MEL-28 cells supplemented with exogenous glutamine, valine, or tyrosine (200 μM). Data are mean ± SEM derived from three independent experiments with * *p <* 0.05, ** *p <* 0.01, and *** *p <* 0.001. Statistical analysis was done using one-way ANOVA with Tukey post-test for multiple comparisons. Scale bar is 100 μm.

## Figure S1

**
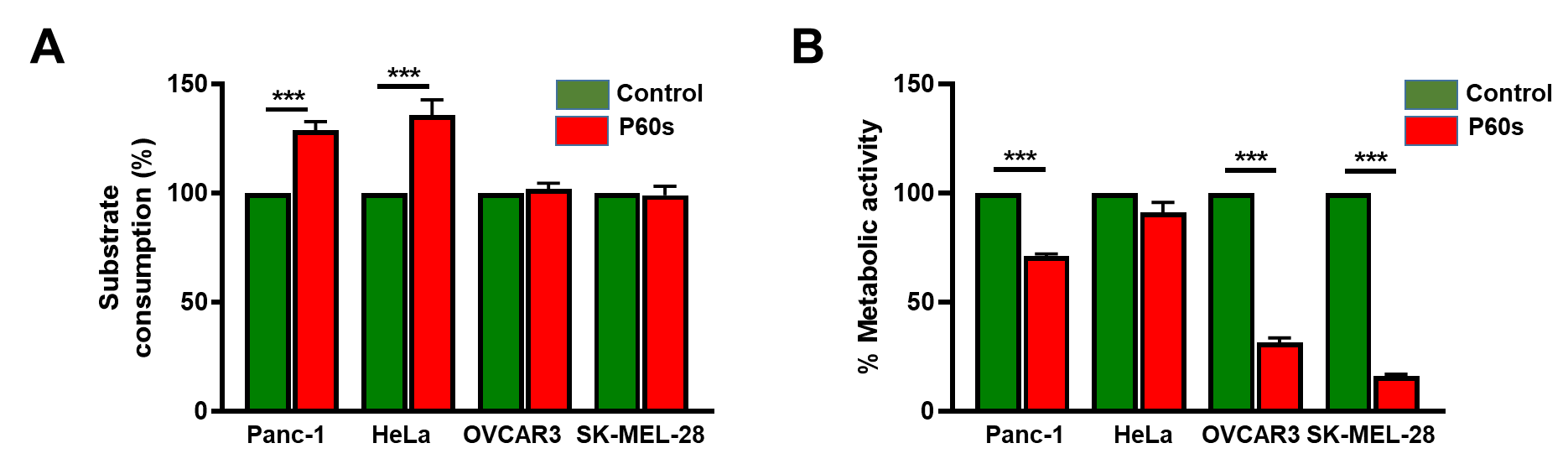
**

## Figure S2

**
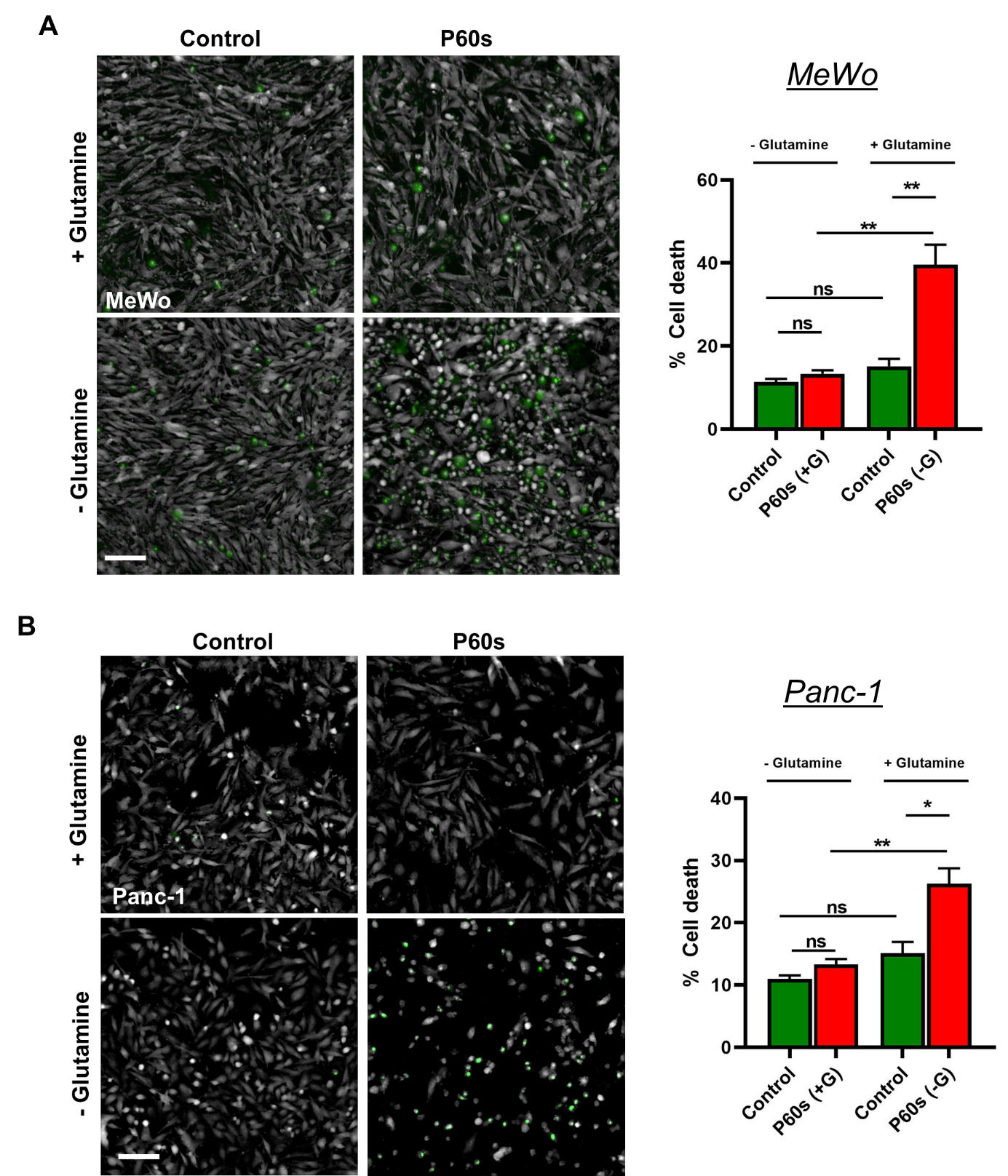
**

## Figure S3


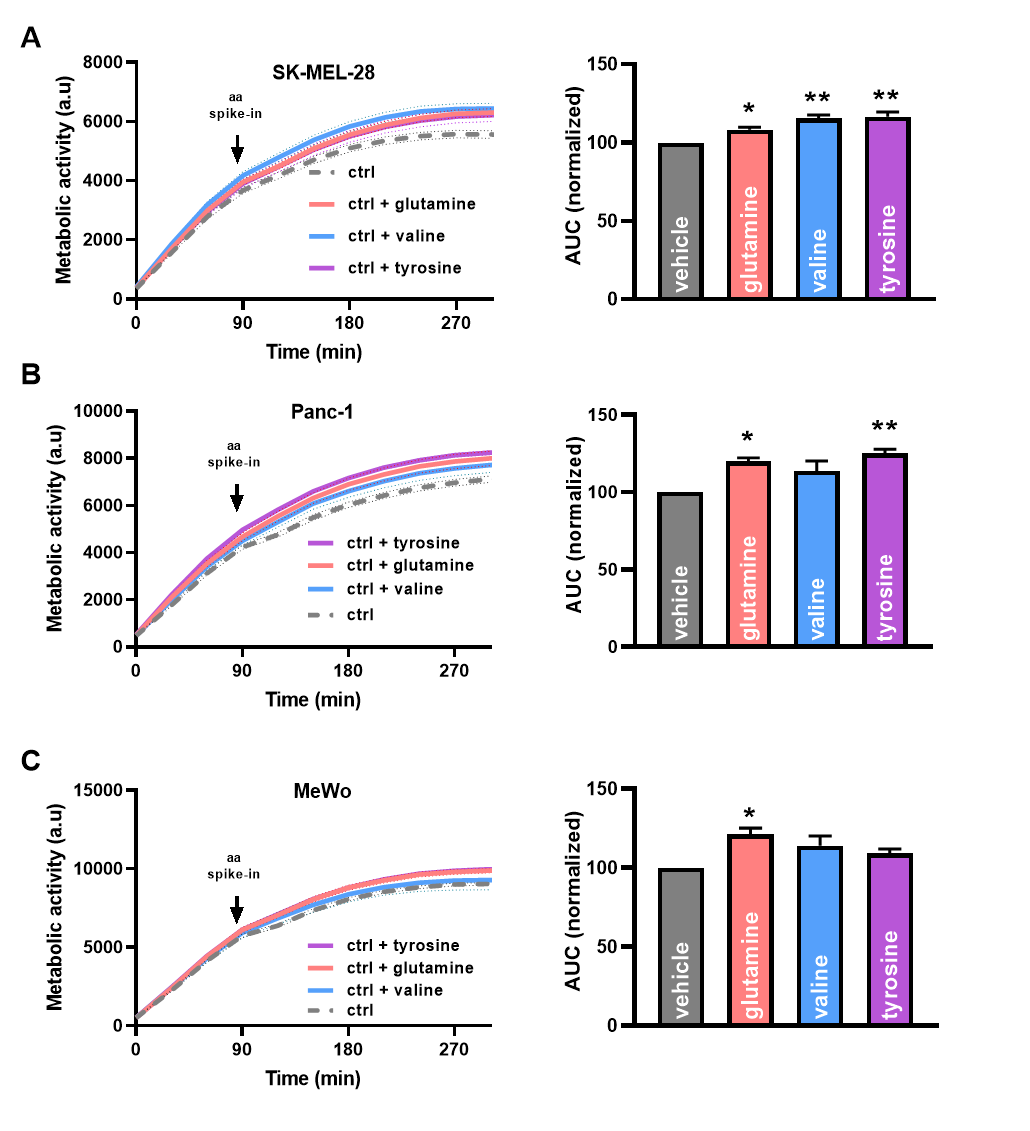


## Figure S4


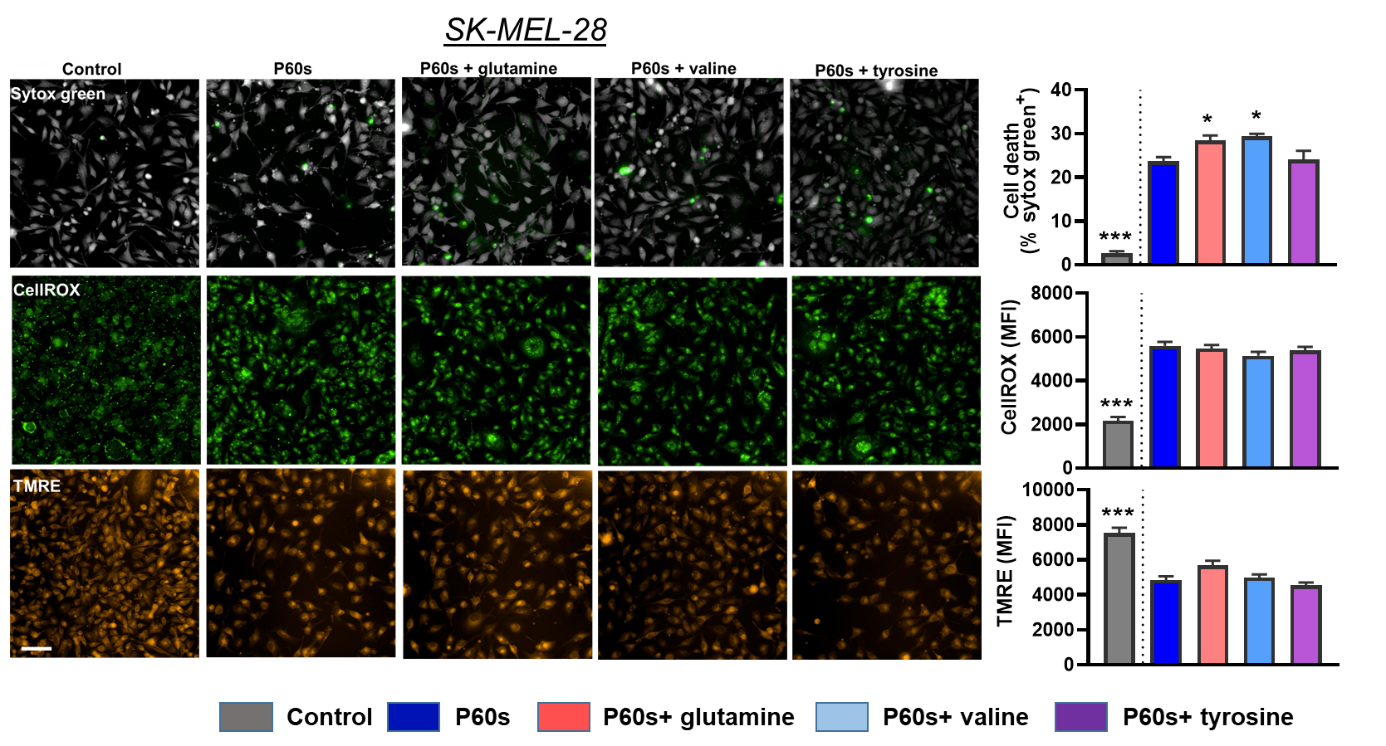

Supplement: Supplementary file 1 — Supplemental material [file 41416_2021_1335_MOESM1_ESM.docx]
